# Therapeutic Effects of Vitamins in Endometriosis Patients: A Systematic Review of Randomized Controlled Trials

**DOI:** 10.3390/ijms27031476

**Published:** 2026-02-02

**Authors:** Sophia Tsokkou, Alkis Matsas, Ioannis Konstantinidis, Evaggelia Karopoulou, Theodora Papamitsou, Eleni Stamoula

**Affiliations:** 1 Department of Medicine, Faculty of Health Sciences, Aristotle University of Thessaloniki, 54124 Thessaloniki, Greece; ikonsc@auth.gr; 2 Laboratory of Experimental Surgery and Surgical Research ‘N.S. Christeas’, Medical School, National and Kapodistrian University of Athens, 11527 Athens, Greece; amatsas@med.uoa.gr; 3 Second Department of Obstetrics and Gynecology, Aretaieion University Hospital, Medical School, National and Kapodistrian University of Athens, 76 Vasilissis Sofias Avenue, 11528 Athens, Greece; karopoulou.eva@gmail.com; 4 Laboratory of Histology-Embryology, Department of Medicine, Faculty of Health Sciences, Aristotle University of Thessaloniki, 54124 Thessaloniki, Greece; thpapami@auth.gr; 5 Department of Clinical Pharmacology, School of Medicine, Aristotle University of Thessaloniki, 54124 Thessaloniki, Greece

**Keywords:** endometriosis, vitamins, micronutrients, antioxidants, vitamin D, vitamin E, vitamin C, randomized controlled trials, pain management, dysmenorrhea, pelvic pain, oxidative stress, inflammation, immunomodulation, nutritional therapy, complementary medicine, reproductive health, women’s health

## Abstract

Endometriosis is a chronic, estrogen-dependent inflammatory condition affecting approximately 10% of women of reproductive age worldwide. It is characterized by the presence of endometrial-like tissue outside the uterine cavity, which frequently results in dysmenorrhea, chronic pelvic pain, dyspareunia, and infertility. While hormonal medications and surgical procedures are common treatments, they are often constrained by adverse effects and high recurrence rates. The aim was to systematically identify, critically appraise, and synthesize randomized controlled trials evaluating vitamin D, C, and E supplementation in women with endometriosis, focusing on their effects on pelvic pain, dysmenorrhea, dyspareunia, quality of life, oxidative and inflammatory biomarkers, and fertility-related outcomes, and to highlight methodological gaps that can inform future research and integrated therapeutic strategies. Following PRISMA guidelines, seven eligible RCTs were identified from databases including PubMed, Scopus, and ScienceDirect. The quality of these studies was assessed using the Jadad Scoring System and Cochrane RoB 2 tool. High-dose supplementation of vitamin D (50,000 IU) was found to significantly reduce pelvic pain and improve biochemical markers such as hs-CRP and total antioxidant capacity (TAC). Vitamin D appears to modulate endometrial pathways by reducing active β-catenin protein activity, which may disrupt signaling associated with lesion invasion and survival. Additionally, combined Vitamin C and E therapy (typically 1000 mg/day of Vitamin C and 800 IU/day of Vitamin E) acts synergistically to scavenge free radicals. This intervention significantly decreased oxidative stress markers, including malondialdehyde (MDA) and reactive oxygen species (ROS). Patients reported significant improvements in symptoms, including a 43% reduction in daily pelvic pain and a 37% reduction in dysmenorrhea. Despite physiological improvements, there was no statistically significant increase in pregnancy rates observed across the trials. Vitamin supplementation with D, C, and E represents a safe, low-cost adjunct therapy that can effectively mitigate endometriosis-related oxidative stress and pelvic pain. While these vitamins show promise for symptom relief, further research with larger sample sizes is required to determine their long-term impact on fertility outcomes and lesion regression.

## 1. Introduction

### 1.1. Definition and Epidemiology

Endometriosis is defined as a chronic, estrogen-dependent inflammatory condition characterized by the presence of endometrial-like tissuee outside the uterine cavity, affecting roughly 10% of women in reproductive age across the globe [1,2]. Ovarian endometriomas are present in approximately 17–44% of women with endometriosis and account for about 35% of benign ovarian cysts requiring surgery [3]. Condition’s symptomatology includes dysmenorrhea, chronic pelvic discomfort, dyspareunia, and infertility, greatly diminishing the quality of life of each woman affected [1]. Even though a substantial body of information is widespread and presents a great advantage to the general population, the pathophysiology of endometriosis is not fully elucidated to date, and existing treatment modalities, mainly hormonal medications and surgical procedures, are frequently constrained by adverse effects, recurrence, and contraindications [4].

### 1.2. Molecular Pathways

The molecular pathways involved in endometriosis are characterized by dysregulated estrogen signaling, progesterone resistance, chronic inflammation, and immune dysfunction. Ectopic endometrial tissue exhibits increased local estradiol production due to upregulated aromatase and steroidogenic acute regulatory protein, with decreased 17β-hydroxysteroid dehydrogenase 2, leading to enhanced estrogen receptor β (ERβ) activity that promotes lesion survival and proliferation [1,5,6,7].

Progesterone resistance is driven by reduced expression and hypermethylation of the progesterone receptor B (PR-B) and its target genes, impairing normal endometrial differentiation and anti-inflammatory responses. Chronic inflammation is sustained by elevated levels of cytokines (e.g., IL-1β, IL-6, and TNF-α), chemokines (CCL2, CCL5), and prostaglandins, with the activation of nuclear factor κB (NF-κB) and MAPK pathways, which further promote angiogenesis and cellular adhesion. Immune dysfunction is evident, with impaired natural killer cell activity and altered macrophage function facilitating immune evasion and persistence of ectopic lesions [1,5,6,7].

**Figure 1:**
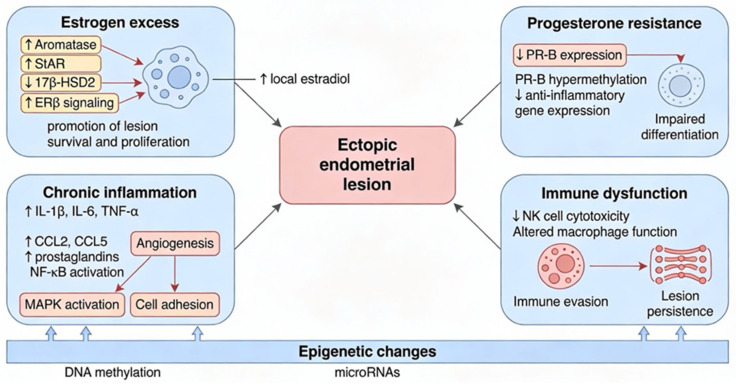
Pathophysiological mechanisms underlying endometriosis: estrogen excess, progesterone resistance, chronic inflammation, and immune dysfunction converge to promote ectopic lesion survival and progression. Epigenetic modifications further exacerbate these pathways, reinforcing the inflammatory and fibrotic nature of the disease.

Epigenetic modifications, including aberrant DNA methylation and altered microRNA expression, further contribute to the pathogenesis by affecting gene expression related to hormone signaling and inflammation. These interconnected pathways underlie the chronic, estrogen-dependent, inflammatory, and fibrotic nature of endometriosis and are targets for emerging therapeutic strategies [1,5,6,7] (Figure 1).

These abnormalities collectively create a microenvironment characterized by chronic inflammation, oxidative stress, and impaired immune surveillance in the peritoneal cavity and ectopic endometrial tissue. Because vitamins D, C, and E have well-described immunomodulatory and antioxidant properties, they are biologically plausible candidates to target several of the pathways outlined above.

### 1.3. Manifestations and Classifications

The manifestations of endometriosis vary from superficial peritoneal lesions of diverse pigmentation to ovarian cysts (endometriomas), nodules with a penetration depth surpassing 5 mm (deep endometriosis, frequently associated with scarring (fibrosis) and adhesions), and extrapelvic lesions [8]. The American Society for Reproductive Medicine (ASRM) has devised a classification of endometriosis-related pain symptoms based on the appearance of peritoneal and pelvic implants, including red, white, and black lesions, with the percentage of involvement for each lesion to be included [9].

Severe endometriosis, as per the revised American Society of Reproductive Medicine (rASRM) staging classification (stages I to IV), does not correlate with symptoms, treatment efficacy, or prognosis [10]. The natural history of endometriosis remains unclear; however, the subphenotypes of lesions may differ during an individual’s life span [1]. Nonetheless, there is no substantial evidence to endorse a systematic progression of endometriotic abnormalities. In analyses of repeat surgeries, lesions advanced in 29% of cases, regressed in 42%, or remained unchanged in 29%, as per rASRM staging; symptom severity or recurrence showed no correlation with stage. Due to the inadequacy of categorization and staging systems in delivering informative clinical algorithms for assessing risk or prognosis, some individuals contend that the definition of symptomatology should encompass probable endometriosis [3,11].

**Figure 2:**
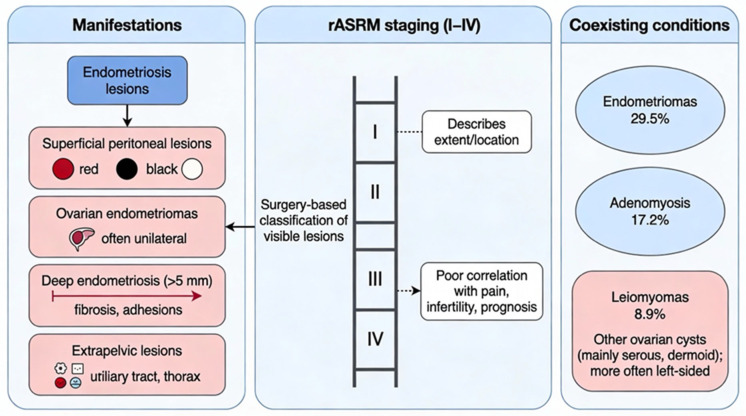
Clinical spectrum and staging of endometriosis: lesion types range from superficial peritoneal implants to deep infiltrating and extrapelvic disease. The rASRM staging system (I–IV) reflects surgical findings but lacks correlation with symptom severity or prognosis. Coexisting gynecological conditions, especially endometriomas, adenomyosis, and leiomyomas, frequently complicate diagnosis and management.

A multicenter study conducted by Matalliotaki Ch. et al. (2017) [12] revealed that among 1000 females with endometriosis that were included in the study, endometriomas were mostly observed as co-existing factors in 29.5% of the cases, followed by adenomyosis and uterine leiomyomas in 17.2 and 8.9%, respectively. The patients with adenomyosis were noted to be of an older age with mean and standard deviation (SD) of 42 ± 3.6 years. When it comes to clinical characteristics, infertility (47.8%) was mostly present in women with endometriomas. Furthermore, adnexal mass (69.4%) was found in patients with ovarian cysts, and pelvic pain was found mostly (53.5%) in women with adenomyosis. In patients with unilateral endometriomas, the observed proportion of left-sided cysts was found in 65.6% of cases, which is significantly greater compared with right-sided cysts—34.4%—and significantly different from the expected incidence of 50% (*p* < 0.001). Additionally, other ovarian cysts in women with endometriosis were detected left-sided in 60% of cases, notably higher compared with right-sided cysts (40%) and significantly different from the expected incidence of 50% (*p* < 0.01). Of note, in the cases with ovarian cysts, the serous cyst was the most common (8.1%), followed by dermoid (1.5%) (Figure 2).

Given the central role of chronic inflammation, oxidative stress, and immune dysregulation in endometriosis pathophysiology, vitamins D, C, and E represent biologically plausible, low-toxicity adjuncts that may modulate these pathways and improve symptoms. However, clinical evidence from randomized controlled trials (RCTs) remains fragmented, with heterogeneous populations, dosing regimens, and outcome measures; no prior systematic review has focused exclusively on RCTs evaluating these vitamins in women with endometriosis.

Therefore, the aim of this systematic review is to identify, critically appraise, and synthesize RCTs investigating vitamin D, C, and E supplementation in endometriosis, with a particular focus on pain, quality of life, inflammatory and oxidative stress biomarkers, and fertility-related outcomes. Clarifying the magnitude, consistency, and methodological robustness of these effects is important for informing clinical practice and for guiding the design of future mechanistic and interventional studies in this field.

#### Objective

Thus, the primary objective of this systematic review is to critically evaluate and synthesize evidence from RCTs investigating the therapeutic effects of vitamin supplementation in patients diagnosed with endometriosis.

## 2. Methods

### 2.1. Registration

**Figure 3:**
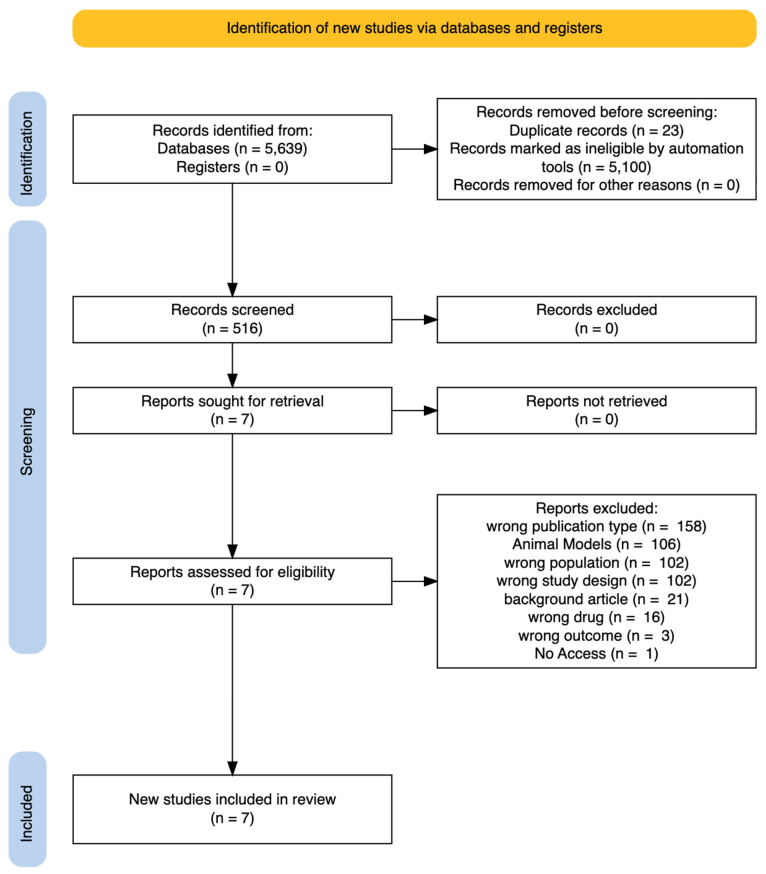
PRISMA flow diagram.

The current systematic review was commenced on the 21 February 2025 and was conducted in accordance with the Preferred Reporting Items for Systematic Reviews and Meta-Analyses (PRISMA) guidelines to ensure transparency and reproducibility (Figure 3 and Supplementary Table S1). The study is registered at PROSPERO with ID CRD420250655809.

### 2.2. Research Aim

The objective of this systematic review is to identify, critically appraise, and synthesize RCTs investigating vitamin supplementation in women with endometriosis, with a specific focus on antioxidant vitamins D, C, and E. It aims to evaluate the effectiveness of these vitamins in mitigating endometriosis-associated symptoms (pelvic pain, dysmenorrhea, and dyspareunia) and improving quality of life, to examine changes in oxidative stress and inflammatory biomarkers (such as plasma malondialdehyde, reactive oxygen species, and cytokine concentrations) following vitamin therapy, to compare outcomes between vitamin-supplemented and placebo or standard-therapy groups in order to determine clinical relevance and statistical significance, and to identify methodological limitations and evidence gaps in existing RCTs that can guide future research directions and support the development of integrated therapeutic strategies for endometriosis care.

### 2.3. Research Question and (PICO) Framework

For the accurate depiction of the research question, the Population, Intervention, Comparison, Outcome (PICO) Framework was used to outline the research question clearly and precisely.

The research question stated is:

“In women with endometriosis, does vitamin supplementation (vitamins D, C, or E), compared with placebo or standard care, reduce endometriosis-related pain and oxidative/inflammatory biomarkers, and does it influence fertility outcomes?” The exact framework is stated in Table 1.

### 2.4. Eligibility Criteria

For a study to be eligible and included in this review, the inclusion and exclusion criteria had to be met, as presented in Table 2.

### 2.5. Screening Process

Following the elimination of articles according to the aforementioned criteria by both automated methods and researchers, the final collection of papers was obtained. To guarantee precision and impartiality, two independent reviewers (I.K. and S.T.) originally evaluated the titles and abstracts through a double-blinded procedure. For research that met the initial criteria, the whole texts were acquired and subsequently assessed to ascertain their ultimate eligibility. Any inconsistencies identified throughout the screening process were addressed by a third reviewer (A.M.).

### 2.6. Charting and Data Extraction

The primary researchers (I.K. and S.T.) systematically extracted key variables from all included studies, including the study ID/author, year, country, sample size, patient demographics, type of vitamin used, dosage and duration, control group details, primary outcomes measured, key findings, and finally, the key findings as a conclusion.

### 2.7. Risk-of-Bias Assessment and Quality Assessment

The methodological quality of the included RCTs was evaluated using the Cochrane Risk of Bias 2 (RoB 2) tool, in addition to the Jadad score. For each trial, the risk of bias was judged using the following five domains: (1) bias arising from the randomization process, (2) bias due to deviations from intended interventions, (3) bias due to missing outcome data, (4) bias in measurement of the outcome, and (5) bias in selection of the reported result, and then derived an overall risk-of-bias judgment. Two reviewers independently performed the RoB 2 assessments, with disagreements resolved by discussion and, when necessary, consultation with a third reviewer. The results are presented in a traffic-light plot summarizing domain-level judgements for all seven RCTs.

## 3. Results

### 3.1. PRISMA-Guided Study Screening and Eligibility Assessment

From a total of 5639 papers initially identified across PubMed (Medline), Scopus, and ScienceDirect databases, a total of seven RCTs were finalized as being appropriate for the inclusion in the study. Out of the initial record, 5100 were excluded from the automation tools and 23 as duplicated records. The rest were excluded in accordance with being the wrong publication type (*n* = 158), animal models (*n* = 106), wrong study design (*n* = 102), wrong population investigated (*n* = 102), background articles (*n* = 21), wrong drug/medication used (*n* = 16), wrong outcome (*n* = 3), and no access (*n* = 1). The PRISMA flow diagram presented in Figure 3 accurately reveals the exact screening process of the study.

### 3.2. Therapeutic Outcomes of Vitamin D in Endometriosis: Evidence from RCTs

Several randomized, placebo-controlled trials have investigated vitamin D supplementation in women with endometriosis using different dosing regimens and clinical settings. High-dose protocols (e.g., 50,000 IU administered weekly or every two weeks) and lower daily doses (e.g., 2000 IU/day) have been evaluated, with generally favorable but not uniformly consistent effects on pain and biochemical markers. Overall, these studies suggest that vitamin D may modulate inflammation and oxidative stress in endometriosis, although the magnitude and robustness of clinical benefit appear to depend on dose, regimen, and study context [13,14,15].

A randomized, double-blind, placebo-controlled trial by Mehdizadehkashi et al. (2021) [13] examined the effect of high-dose vitamin D in women with endometriosis-related pain after laparoscopic diagnosis. Participants received 50,000 IU of vitamin D orally every 2 weeks for 12 weeks or a matching placebo. The intervention group showed reductions in pelvic pain scores and improvements in biochemical indices, including lower hs-CRP levels and increased total antioxidant capacity, together with changes in lipid parameters. These findings are in line with vitamin D’s proposed anti-inflammatory and antioxidative actions in endometrial tissue.

Pazhohan et al. (2021) [14] focused on molecular outcomes by evaluating β-catenin in infertile women with stage III–IV endometriosis. In this exploratory randomized trial, patients received 50,000 IU of vitamin D weekly for 12–14 weeks in addition to routine care, or routine care alone. Vitamin D supplementation was associated with a reduction in active β-catenin protein in endometrial tissue without significant changes in gene expression, suggesting a post-translational regulatory effect on the Wnt/β-catenin pathway. This supports a potential mechanism through which vitamin D may influence endometrial receptivity and lesion behavior.

Nodler et al. (2020) [15] investigated adolescents and young women with surgically confirmed endometriosis and chronic pelvic pain in a three-arm, double-blind trial comparing daily vitamin D3 (2000 IU), fish oil, and placebo over six months. Pain scores decreased over time in all groups, and while vitamin D supplementation produced a statistically significant within-group reduction in VAS pain, the improvement was similar in magnitude to that observed with placebo. This substantial placebo response underscores the influence of non-specific trial factors and clinical follow-up on subjective pain outcomes in this population.

The double-blind RCT by Almassinokiani et al. (2016) [16] investigated the effect of vitamin D supplementation on pain associated with endometriosis after laparoscopic surgery. The results indicated no statistically significant differences in pain alleviation between the vitamin D and placebo cohorts at 24 weeks following surgery. However, the outcome may be attributed to several factors including the limited sample size, the absence of baseline vitamin D level evaluation, and the possibility that surgical intervention alone may have significantly alleviated discomfort. Notwithstanding encouraging outcomes in preclinical investigations, the findings indicate that vitamin D supplementation may not provide significant advantages in post-surgical pain treatment for endometriosis. These results must be further backed up by large sample size RCTs.

Taken together, these RCTs indicate that vitamin D supplementation can favorably influence inflammatory and oxidative stress markers and may reduce endometriosis-related pain in some settings, particularly with high-dose regimens. However, inconsistent effects on clinical symptoms across trials, heterogeneous dosing schedules, and methodological limitations (including small sample sizes and variable baseline vitamin D status) mean that the overall evidence for routine vitamin D use in endometriosis remains suggestive rather than definitive. Larger, well-powered trials with standardized dosing are needed to clarify the therapeutic role of vitamin D in this condition [13,14,15,16].

### 3.3. Effects of Antioxidant Supplementation (Vitamins C and E) on Oxidative Stress and Clinical Outcomes

Furthermore, a triple-blind RCT conducted by Amini et al. (2021) [17] substantiates the therapeutic efficacy of antioxidant supplementation, particularly in relation to vitamins C and E, in diminishing oxidative stress indicators and mitigating endometriosis-related pain. The results demonstrated a significant decrease in malondialdehyde (MDA) and reactive oxygen species (ROS) levels, along with notable improvements in dysmenorrhea, dyspareunia, and chronic pelvic pain assessments following eight weeks of supplementation. Even though the total antioxidant capacity (TAC) remained unchanged, the study presented improvements in symptomatology strongly suggest that targeted reduction in oxidative stress markers, rather than systemic antioxidant capacity per se, may be meaningful clinical outcomes in women who have endometriosis. Biochemical interaction among the two vitamins is recognized for its synergistic free-radical scavenging activities, appearing to highlight the significance of co-supplementation strategies in altering inflammatory pathways associated with pelvic pain.

Similarly, the RCT trial by Santanam et al. (2013) [18], providing evidence for the efficacy of antioxidant therapy in relation to vitamins C and E, is presented. Specifically, following an eight-week supplementation regimen, observable improvements were noted in pain-related metrics, encompassing a 43% decrease in daily pelvic pain, a 37% reduction in dysmenorrhea, and a 24% decline in dyspareunia. Moreover, inflammatory markers in the peritoneal fluid, specifically RANTES, IL-6, and MCP-1, were significantly reduced, indicating a biochemical mechanism that supports clinical advantages.

In another double-blind RCT study, Mier-Cabrera et al. (2008) [19] evaluated the effects of vitamin C and E supplementation on oxidative stress markers and pregnancy outcomes in women with stage I–II endometriosis. Participants were provided with either a placebo or a daily antioxidant bar containing high doses of vitamin C (343 mg) and vitamin E (84 mg) during a period of six months. The results showed a notable decrease in peripheral malondialdehyde (MDA) levels by month four and lipid hydroperoxides (LOOHs) by month six in the vitamin group relative to the placebo, thereby validating the intervention’s antioxidant effectiveness. No substantial rise in pregnancy rates was observed, indicating that although antioxidant supplementation may reduce oxidative stress, its effect on fertility outcomes in endometriosis has not been demonstrated in the available trials.

The vitamin supplementation from the seven RCTs exhibited favorable outcomes in mitigating symptoms related to endometriosis, especially pelvic pain and oxidative stress. High-dose vitamin D interventions, exemplified by the studies of Mehdizadehkashi et al. [13] and Pazhohan et al. [14], yielded significant reductions in pelvic pain, inflammatory markers such as hs-CRP, and molecular alterations including diminished β-catenin activity; however, some investigations, including those by Almassinokiani et al., did not report considerable improvement following surgery.

The incorporation of antioxidant vitamins C and E demonstrated similar advantages across trials, with research by Amini et al. [17] and Santanam et al. [18] indicating substantial decreases in pain metrics and oxidative indicators, including MDA and ROS, as well as reduced cytokine levels. Mier-Cabrera et al. [19] emphasized antioxidant effects; however, reproductive outcomes showed no statistical enhancement. These therapies were generally well-tolerated, with no significant adverse effects noted, thus emphasizing the possible therapeutic function of vitamins in the therapy of endometriosis.

### 3.4. Interpretation of Jadad Scale Quality Assessment

The tables presented in Table 3 and Table 4 present the quality assessment performed using the Jadad Scoring Scale.

Across all studies, randomization was consistently reported; however, the rigor and transparency of the randomization method varied substantially. Only a subset of trials such as Nodler et al. (2020) [15] and Mehdizadehkashi et al. (2021) [13] provided clear descriptions of their randomization techniques. In contrast, several studies mentioned randomization without detailing the method, which limited their methodological score.

Blinding practices also differed notably. While most trials implemented some form of blinding, the degree and appropriateness of blinding ranged from absent (Pazhohan, 2021 [14]) to rigorous triple-blinding (Amini, 2021 [17]). Studies employing well-matched placebos or identical coding achieved higher scores, whereas those lacking clear descriptions of blinding procedures or placebo matching received lower ratings.

Reporting of withdrawals and dropouts was generally adequate, with most studies documenting participant attrition and providing reasons. This transparency contributed positively to the overall quality scores.

Overall, the quality assessment indicates that while several included studies demonstrate robust methodological design, others exhibit reporting limitations that may influence the reliability of their findings. These variations should be considered when interpreting the collective evidence on vitamin D and antioxidant supplementation in endometriosis.

### 3.5. Interpretation of Cochrane RoB 2 Tool

Using the Cochrane RoB 2 tool, all seven RCTs were judged at low risk of bias for deviations from intended interventions, missing outcome data, and measurement of outcomes, reflecting predominantly double- or triple-blind designs, minimal attrition, and the use of validated pain scales and laboratory biomarkers (Table 5 and Table 6).

In contrast, some concerns were frequently identified in the randomization process (four trials with incompletely described sequence generation and/or allocation concealment) and in selection of the reported result, as none of the trials had a publicly available preregistered protocol or analysis plan.

Overall, three studies (Nodler 2020 [15], Mehdizadehkashi 2021 [13], and Amini 2021 [17]) were rated as low risk of bias, whereas four (Pazhohan 2021 [14], Santanam 2013 [18], Almassinokiani 2016 [16], and Mier-Cabrera 2008 [19]) were rated as having some concerns, and no trial was classified as high risk of bias.

Jadad scores were generally consistent with these findings, indicating low-to-moderate trial quality, but they did not capture the domain-specific issues highlighted by the RoB 2 assessment. Table 7 and Table 8 are synthesized based on the most important findings of each study included.

## 4. Discussion

### 4.1. Vitamin D and Endometriosis

#### 4.1.1. Modulator of Endometrial Molecular Pathways

Vitamin D acts as a modulator of endometrial molecular pathways in endometriosis primarily through its anti-inflammatory, anti-proliferative, and immunomodulatory effects. The active form, 1,25-dihydroxyvitamin D3 (1,25[OH]_2_D_3_), binds to the vitamin D receptor (VDR) expressed in endometrial stromal cells, leading to suppression of pro-inflammatory cytokine production (e.g., IL-8, TNF-α), inhibition of NF-κB signaling, and reduction in prostaglandin synthesis, which collectively dampen the chronic inflammatory milieu characteristic of endometriosis [20,21,22,23].

Vitamin D also downregulates the expression of matrix metalloproteinases (MMP-2, MMP-9), growth factors (EGF, PDGFB, and MDGF), and cell adhesion molecules such as CD44, thereby reducing cellular proliferation, invasion, and migration of endometriotic cells. In addition, vitamin D modulates the Wnt/β-catenin pathway, decreasing β-catenin activity and CD44 expression, which are implicated in the enhanced invasiveness and survival of endometriotic lesions [14,22,24].

**Figure 4:**
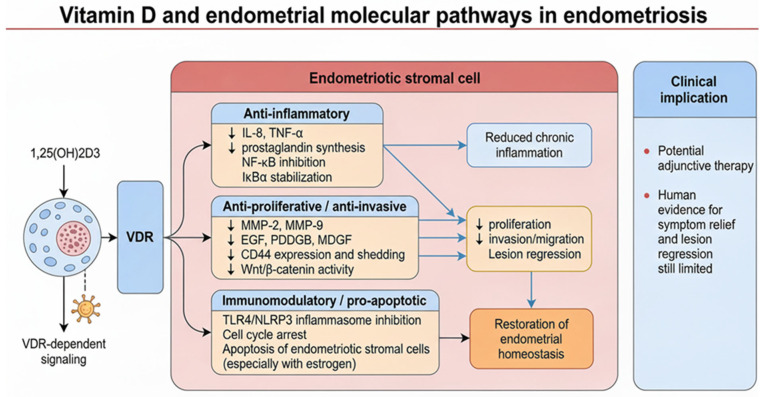
Vitamin D and endometrial molecular pathways in endometriosis: through VDR-dependent signaling, 1,25(OH)_2_D_3_ exerts anti-inflammatory, anti-proliferative, and immunomodulatory effects on endometriotic stromal cells. These mechanisms include suppression of NF-κB, inhibition of MMPs and growth factors, and promotion of apoptosis, collectively contributing to lesion regression and potential symptom relief.

Furthermore, vitamin D promotes cell cycle arrest and induces apoptosis in endometriotic stromal cells, especially in the presence of estrogen, contributing to lesion regression [7]. These molecular actions are VDR-dependent and result in the stabilization of IκBα, inhibition of TLR4/NLRP3 inflammasome activation, and restoration of endometrial homeostasis [22,25,26] (Figure 4).

Collectively, these mechanisms position vitamin D as a potential adjunctive therapeutic agent in endometriosis, although clinical efficacy in symptom relief and lesion regression remains to be fully established in human studies.

#### 4.1.2. Clinical Evidence for Vitamin D in Endometriosis Management

The clinical evidence for vitamin D supplementation in endometriosis presents mixed results, with some trials demonstrating benefits in pain reduction and inflammatory markers while others show no significant advantage over placebo.

High-dose vitamin D (50,000 IU) supplementation has shown the most consistent benefits in clinical trials. The Mehdizadehkashi et al. (2021) [13] trial demonstrated that 12 weeks of 50,000 IU vitamin D biweekly significantly reduced pelvic pain (mean difference −1.12; 95% CI, −2.1, −0.09; and *p* = 0.03), decreased hs-CRP by 0.64 mg/L (*p* < 0.001), and increased total antioxidant capacity by 47.54 mmol/L (*p* = 0.001) compared to placebo [13]. These findings support vitamin D’s anti-inflammatory and antioxidative mechanisms in endometriosis pathophysiology.

The Pazhohan et al. (2021) [14] study provides crucial insight, demonstrating that 50,000 IU weekly vitamin D for 12–14 weeks significantly reduced active β-catenin protein in endometrial tissue of women with stage III/IV endometriosis without altering mRNA expression, suggesting post-translational regulation of the Wnt/β-catenin pathway [24]. This is particularly relevant given that overactive Wnt/β-catenin signaling is associated with reduced endometrial receptivity and implantation failure in endometriosis. The same research group showed that vitamin D supplementation decreased CD44 expression and shedding, another Wnt target gene involved in endometrial invasion and adhesion.

However, lower-dose vitamin D (2000 IU daily) has shown less-convincing results. The Nodler et al. (2020) [15] SAGE trial in adolescents and young women found that while vitamin D supplementation led to statistically significant VAS pain reduction from 7.0 to 5.5 (*p* = 0.02), the placebo group experienced nearly identical improvement (6.0 to 4.4, *p* = 0.07), with no significant difference between groups. This substantial placebo effect highlights the importance of non-specific trial factors and clinical engagement in pain management.

Post-surgical vitamin D supplementation has not demonstrated clear benefits. The Almassinokiani et al. (2016) [16] trial showed no significant differences in pain relief between vitamin D and placebo groups at 24 weeks post-laparoscopy. This may reflect that surgical intervention itself provides substantial pain relief, potentially masking any additional vitamin D benefit, or that the study was underpowered with inadequate baseline vitamin D assessment.

Meta-analyses reveal important limitations. A 2022 systematic review found no significant effect of vitamin D on dysmenorrhea (mean −0.71; 95% CI −1.94, 0.51) or non-cyclic pelvic pain (mean 0.34; 95% CI −0.02, 0.71) when pooling human studies, despite promising in vitro and animal data showing lesion regression and decreased invasion [5]. However, a 2023 meta-analysis of antioxidant vitamins found that while vitamin E/C combinations effectively reduced endometriosis-related pain, vitamin D supplementation showed a trend toward pain reduction that did not reach statistical significance compared to placebo [27].

Baseline vitamin D status appears important. Serum 25-hydroxyvitamin D3 levels are significantly lower in women with severe endometriosis compared to controls and those with mild disease [23]. Population data from NHANES showed an inverse correlation between adequate vitamin D levels and endometriosis risk (OR 0.73; 95% CI 0.54–0.97) [28]. This suggests that vitamin D deficiency may be a risk factor for disease severity, though causality remains unestablished.

The heterogeneity in dosing regimens (ranging from 2000 IU daily to 50,000 IU weekly), treatment duration (6–24 weeks), patient populations (adolescents vs. adults; post-surgical vs. medical management), and outcome measures limits definitive conclusions. The absence of baseline vitamin D level stratification in most trials prevents assessment of whether supplementation benefits are restricted to vitamin D-deficient patients. Future research should focus on adequately powered trials with standardized dosing, baseline vitamin D assessment, longer follow-up periods, and objective endpoints including lesion regression and fertility outcomes to clarify vitamin D’s therapeutic role in endometriosis management [21,23,27,29].

### 4.2. Vitamins C and E and Endometriosis

#### 4.2.1. Clinical Effects of Vitamins C and E in Endometriosis

**Figure 5:**
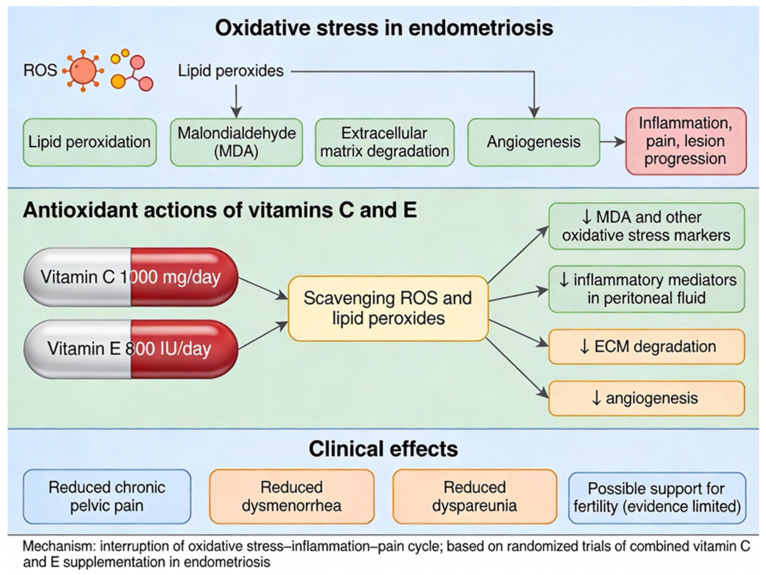
Oxidative stress and antioxidant therapy in endometriosis: reactive oxygen species drive lipid peroxidation, inflammation, and lesion progression. Vitamins C and E interrupt this cycle by scavenging ROS and reducing MDA, ECM degradation, and angiogenesis, leading to measurable improvements in pelvic pain and inflammatory markers.

Vitamins C and E modulate these molecular pathways by acting as potent antioxidants. They neutralize ROS and lipid peroxides, reducing oxidative damage and interrupting the feed-forward cycle of inflammation and pain. Clinical evidence demonstrates that combined supplementation with vitamin C (1000 mg/day) and vitamin E (800 IU/day) significantly decreases oxidative stress markers (e.g., malondialdehyde), lowers inflammatory mediators in peritoneal fluid, and alleviates chronic pelvic pain, dysmenorrhea, and dyspareunia in endometriosis patients [30,31,32]. These vitamins also inhibit extracellular matrix degradation and angiogenesis, supporting lesion stabilization and potentially improving fertility outcomes [30,33,34] (Figure 5).

#### 4.2.2. The Role of Vitamins C and E in Endometriosis Management

The synergistic combination of vitamins C and E demonstrates significant therapeutic efficacy in reducing oxidative stress markers and alleviating endometriosis-associated pain through complementary antioxidant mechanisms. The triple-blind RCT by Amini et al. (2021) [17] provides robust evidence that eight weeks of combined supplementation with vitamin C (1000 mg/day) and vitamin E (800 IU/day) significantly decreased malondialdehyde (MDA) and reactive oxygen species (ROS) levels compared to placebo, with concurrent improvements in dysmenorrhea, dyspareunia, and chronic pelvic pain (all *p* < 0.001) [17]. Although total antioxidant capacity (TAC) remained unchanged, the targeted reduction in specific oxidative stress markers appears to be meaningful clinical benefits, suggesting that the direct scavenging of lipid peroxides and free radicals, rather than systemic antioxidant enhancement, drives therapeutic efficacy [17,35].

The biochemical synergy between vitamins C and E is well-established: vitamin E (α-tocopherol) functions as the primary lipid-soluble antioxidant in biological membranes, scavenging lipid hydroperoxyl radicals, while vitamin C (ascorbate) regenerates vitamin E from its oxidized tocopheroxyl radical form, thereby sustaining continuous antioxidant protection [36,37,38]. This regenerative cycle is particularly relevant in endometriosis, where peritoneal fluid exhibits elevated oxidatively modified lipoproteins that generate pain-inducing eicosanoids and prostaglandins [6]. Meta-analyses confirm that co-administration of vitamins C and E significantly reduces plasma MDA (WMD: −0.38 µg/L, *p* < 0.001) and lipid peroxides (WMD: −1.01 µg/L, *p* < 0.001) while increasing TAC (WMD: 0.09 mmol/L, *p* < 0.001) and glutathione peroxidase activity [39].

The RCT by Santanam et al. (2013) [18] corroborates these findings, demonstrating that eight weeks of vitamin C and E supplementation resulted in a 43% reduction in daily pelvic pain, 37% decrease in dysmenorrhea, and 24% decline in dyspareunia [18]. Critically, this study also documented significant reductions in inflammatory markers within peritoneal fluid, including RANTES, IL-6, and MCP-1, providing evidence that antioxidant therapy attenuates the inflammatory cascade underlying endometriosis-associated pain [2]. The inverse correlation between plasma MDA levels and both duration and dose of vitamin C/E supplementation further supports a dose-dependent therapeutic relationship [31,36,37,39]. Beyond cytokines, several peritoneal fluid proteins related to innate immunity and redox balance have also been implicated in endometriosis. Vitamin D-binding protein (VDBP) and lactoferrin (Lf) are of particular interest: both are involved in neutrophil activation, iron handling, and modulation of local inflammatory responses. Studies evaluating VDBP and Lf in paired plasma and peritoneal fluid samples have shown disease-specific associations between their concentrations and correlations across compartments, suggesting that these proteins may participate in shaping the pro-oxidative and pro-inflammatory peritoneal milieu characteristic of endometriosis and could contribute to biomarker panels for identifying high-risk patients. Although these proteins were not targeted by vitamin interventions in the RCTs included in our review, their interaction with vitamin D signaling and oxidative stress pathways further supports the biological plausibility of micronutrient-based adjunctive strategies in endometriosis [40].

However, the impact of antioxidant supplementation on fertility outcomes remains equivocal. The double-blind RCT by Mier-Cabrera et al. (2008) [19] evaluated six months of supplementation with vitamin C (343 mg/day) and vitamin E (84 mg/day) in women with stage I–II endometriosis, demonstrating significant reductions in peripheral MDA by month four and lipid hydroperoxides by month six [19]. Despite these favorable oxidative stress improvements, no significant increase in pregnancy rates was observed, suggesting that while antioxidant therapy mitigates oxidative damage, its effects on reproductive outcomes may require longer treatment duration, higher doses, or multi-nutrient combinations to achieve clinically meaningful fertility benefits [19,30,41].

The typical dosing regimen across successful trials has been vitamin C 1000 mg/day and vitamin E 800 IU/day for 8–12 weeks, though some studies have used lower doses (vitamin C 343 mg/day; vitamin E 84 mg/day) with more modest effects. The interventions are generally well-tolerated with minimal adverse effects, primarily limited to occasional gastrointestinal disturbances [17,19].

The molecular mechanisms underlying pain relief extend beyond simple ROS neutralization. Oxidative stress in endometriosis activates key proliferation pathways including Raf/MEK/ERK and mTOR, promotes extracellular matrix degradation via matrix metalloproteinases, drives angiogenesis through VEGF upregulation, and sustains neurogenic inflammation through macrophage activation and prostaglandin synthesis. By interrupting these oxidative stress-dependent pathways, vitamins C and E may limit lesion progression while simultaneously alleviating pain symptoms. The observation that oxidatively modified lipoproteins in peritoneal fluid directly induce nociception in animal models, which is suppressed by vitamin E and N-acetylcysteine, provides compelling support for antioxidant therapy in pain management [41,42,43,44].

Despite these promising results, several limitations warrant consideration. The heterogeneity in study designs, including variations in disease stage (stage I–II versus III–IV), baseline vitamin status, outcome measures, and follow-up duration, limits definitive conclusions about optimal treatment protocols [30]. Most trials have focused on short-term pain outcomes (8–24 weeks) without assessing long-term effects on lesion regression, disease recurrence, or quality of life [45]. Furthermore, the lack of baseline vitamin C and E level assessment in most studies prevents determination of whether benefits are restricted to deficient populations or represent universal therapeutic effects [30,42,45].

Future research should prioritize adequately powered, long-term RCTs with standardized dosing protocols, baseline nutritional assessment, and comprehensive outcome measures including lesion size, recurrence rates, fertility parameters, and quality of life indices to establish evidence-based guidelines for antioxidant vitamin supplementation in endometriosis management [30,41,45].

#### 4.2.3. Mechanistic Rationale for Vitamin D, C, and E in Endometriosis

The pathophysiological features summarized in Section 1.2, namely dysregulated estrogen signaling, progesterone resistance, chronic inflammation, oxidative stress, and immune dysfunction, provide a direct rationale for considering vitamins D, C, and E as adjunctive therapies in endometriosis. Vitamin D, via the vitamin D receptor expressed in endometrial and immune cells, downregulates pro-inflammatory cytokines (e.g., IL-6, TNF-α), inhibits NF-κB activation, modulates the Wnt/β-catenin pathway, and can reduce expression of matrix metalloproteinases and adhesion molecules, thereby potentially limiting lesion invasion and sustaining a less inflammatory peritoneal milieu. Vitamins C and E, in contrast, act primarily as antioxidant scavengers of reactive oxygen species and lipid peroxides, interrupting the oxidative stress loop that amplifies pain and inflammation; this is consistent with observed reductions in malondialdehyde, ROS, and peritoneal cytokines (RANTES, IL-6, and MCP-1) in trials of combined vitamin C/E supplementation. By targeting inflammatory and oxidative pathways that are central to endometriosis pathogenesis, these vitamins offer a mechanistically coherent, low-toxicity strategy to complement standard hormonal or surgical treatments, although current RCTs remain small and heterogeneous and do not yet establish causality.

#### Limitations and Prospective Views

This systematic review has several limitations that should be considered when interpreting its findings. First, only seven RCTs met the inclusion criteria, and most had small sample sizes, which limits statistical power and the precision of effect estimates. Second, we restricted inclusion to English-language, peer-reviewed publications, raising the possibility of language and publication bias. Third, substantial heterogeneity in vitamin formulations, dosages, treatment durations, comparator arms, and outcome measures precluded a formal meta-analysis and limited direct comparability across studies. Fourth, all data were extracted from published reports without access to individual participant-level data, which may obscure important subgroup effects (e.g., by baseline vitamin status, disease stage, or concomitant therapies). Finally, although we followed a predefined protocol and used standardized tools for quality and risk-of-bias assessment, subjective judgment in study selection, data extraction, and interpretation cannot be entirely excluded.

From a prospective perspective, future research should prioritize adequately powered, multicenter randomized trials that standardize vitamin D, C, and E dosing regimens, treatment duration, and core outcome sets, including pain, quality of life, biomarker, and fertility endpoints. In addition, preregistered protocols, individual participant data meta-analyses, and stratification by baseline vitamin status, disease severity, and concomitant hormonal or surgical treatments will be essential to clarify which subgroups of women with endometriosis are most likely to benefit from vitamin supplementation and to inform personalized, evidence-based adjunctive therapy.

## 5. Conclusions

In conclusion, the RCTs suggest that vitamin D, C, and E supplementation can reduce endometriosis-related pain and oxidative stress markers, while evidence for improvement in fertility outcomes remains limited and inconclusive. Within these constraints, vitamin supplementation appears to be a generally well-tolerated adjunctive approach that may offer symptomatic benefit for women with endometriosis, although more robust data are needed before firm clinical recommendations can be made.

## Figures and Tables

**Table 1:** PICO framework.

P (Population)	Women of reproductive age (18–50 years) with confirmed diagnosis of endometriosis (by laparoscopy, imaging, or clinical criteria).
I (Intervention)	Vitamin supplementation with vitamins D, C, or E, alone or in combination (any dose, any duration).
C (Comparison)	Placebo, no intervention, or standard medical care (e.g., hormonal therapy, surgical intervention).
O (Outcome)	Primary outcomes: reduction in pain scores (dysmenorrhea, chronic pelvic pain, and dyspareunia); improvement in quality of life; and changes in oxidative stress markers (MDA, ROS) and inflammatory biomarkers (IL-6, CRP, TNF-α). Secondary outcomes: fertility-related measures (pregnancy rate, time to pregnancy) when reported.

**Table 2:** Eligibility criteria.

Category	Inclusion Criteria	Exclusion Criteria
Study Design	Randomized controlled trials (RCTs), including parallel or crossover designs	Non-randomized studies, observational studies, case reports, and reviews
Population	Females of reproductive age with a confirmed diagnosis of endometriosis	Studies involving animals, men, postmenopausal women, or those without confirmed diagnosis
Intervention	Supplementation with vitamins (e.g., vitamin D, C, E, A, or multivitamin combinations) alone or in combination	Non-vitamin interventions, herbal remedies, or dietary patterns without vitamin focus
Comparator	Placebo, no intervention, or conventional medical therapy	Studies without a control group or inappropriate comparator
Outcomes	Pain relief, inflammatory marker changes, oxidative stress markers, and quality of life	Studies reporting only fertility outcomes without assessment of pain, quality of life, or biochemical markers were excluded
Language	Full-text articles published in English	Articles published in languages other than English
Publication Type	Peer-reviewed journals	Abstracts, conference proceedings, letters, or editorials

**Table 3:** Jadad Scale summary—seven RCTs on antioxidants in endometriosis.

Criteria	Pazhohan (2021) [14]	Nodler (2020) [15]	Santanam (2013) [18]	Mehdizadehkashi (2021) [13]	Almassinokiani (2016) [16]	Amini (2021) [17]	Mier-Cabrera (2008) [19]
Randomization described	Yes	Yes	Yes	Yes	Yes	Yes	Yes
Method of randomization	Not explained	Permuted blocks	Not detailed	Computer-generated	Not detailed	Simple random	Not detailed
Blinding described	No	Double-blind	Placebo-controlled	Double-blind	Double-blind	Triple-blind	Double-blind
Blinding method appropriate	Not discussed	Capsules matched	Unclear matching	Capsules matched	Not explained	Identical coding	Bars matched in taste/appearance
Withdrawals/dropouts reported	One dropout	Yes, detailed	Reported	Five withdrawals	One pregnancy-related	Criteria stated	Two dropouts noted

**Table 4:** Jadad Scale—scoring outcome.

	Score	Quality Rating
Pazhohan et al. [14]	2/5	Low quality
Nodler et al. [15]	5/5	High quality
Santanam et al. [18]	3/5	Moderate quality
Mehdizadehkashi et al. [13]	5/5	High quality
Almassinokiani et al. [16]	3/5	Moderate quality
Amini et al. [17]	5/5	High quality
Mier-Cabrera et al. [19]	4/5	High quality

**Table 5:** Risk-of-bias assessment of the seven randomized controlled trials of vitamin supplementation in endometriosis using the Cochrane RoB 2 tool, showing judgments for each bias domain and overall risk of bias.

Study (Year)	Main Outcome Assessed for RoB	Randomization Process	Deviations from Intended Interventions	Missing Outcome Data	Measurement of the Outcome	Selection of Reported Result	Overall RoB
Pazhohan 2021 [14]	Molecular outcome (active β-catenin; vitamin D vs. routine care)	Some concerns (randomization mentioned, method not fully described, no explicit allocation concealment)	Low risk (no clear protocol deviations; interventions well-defined)	Low risk (minimal exclusions; reasons reported)	Low risk (biochemical/molecular lab assays, outcome assessors unlikely to be influenced by knowledge of group)	Some concerns (no published protocol; limited detail on whether all prespecified outcomes were reported)	Some concerns
Nodler 2020 [15]	Pelvic pain (VAS; vitamin D3/fish oil/placebo)	Low risk (permuted block randomization described; clear randomization procedure)	Low risk (double-blind, matched capsules; no major deviations from assigned interventions)	Low risk (attrition described and balanced; reasons provided)	Low risk (validated pain scales; identical procedures across arms; blinding maintained)	Some concerns (no detailed protocol/public registration for specific outcomes; selective reporting cannot be fully ruled out)	Low risk
Santanam 2013 [18]	Pelvic pain and peritoneal cytokines (vitamin C + E vs. placebo)	Some concerns (randomization reported but method not fully described; allocation concealment unclear)	Low risk (placebo-controlled, double-blind; no evidence of systematic deviations)	Low risk (dropouts described; outcome data largely complete)	Low risk (standardized VAS and ELISA measurements; assessors likely blinded)	Some concerns (no explicit protocol; possibility of selective emphasis on significant outcomes)	Some concerns
Mehdizadehkashi 2021 [13]	Pelvic pain, hs-CRP, TAC (vitamin D vs. placebo)	Low risk (computer-generated randomization described; allocation approach reasonably clear)	Low risk (double-blind, matched placebo; no major deviations)	Low risk (reported completion rates; withdrawals minimal and balanced)	Low risk (validated pain scales and lab markers; blinding maintained)	Some concerns (no registered protocol referenced; unclear if all outcomes analyzed were prespecified)	Low risk
Almassinokiani 2016 [16]	Post-operative pain (VAS; vitamin D vs. placebo)	Some concerns (randomization mentioned, but method and allocation concealment not clearly described)	Low risk (double-blind, placebo-controlled; no notable deviations)	Low risk (follow-up described; attrition limited)	Low risk (VAS measurements; likely blinded outcome assessment)	Some concerns (no accessible protocol; negative primary result reported, but selective reporting cannot be fully assessed)	Some concerns
Amini 2021 [17]	Pain and oxidative stress markers (vitamin C + E vs. placebo)	Low risk (randomization with clear description and triple-blinding)	Low risk (triple-blind design reduces risk of deviations; adherence procedures described)	Low risk (dropouts minimal and documented)	Low risk (VAS and biochemical markers with blinded lab assessment)	Some concerns (no detailed preregistered analysis plan cited)	Low risk
Mier-Cabrera 2008 [19]	Oxidative stress markers and pregnancy (vitamin C + E bar vs. placebo bar)	Some concerns (randomization and allocation procedures not fully detailed; older trial)	Low risk (double-blind, matched bars; no evidence of systematic deviations)	Low risk (small sample but attrition reported; pregnancy outcome analyzed on available data)	Low risk (laboratory assays and objective pregnancy outcome; blinding likely preserved)	Some concerns (no protocol; high risk of unreported negative or exploratory outcomes cannot be ruled out)	Some concerns

**Table 6:** Risk-of-bias assessment for the seven randomized controlled trials of vitamin supplementation in endometriosis using the Cochrane RoB 2 tool, showing judgements for randomization, deviations from intended interventions, missing outcome data, measurement of outcomes, selection of reported results, and overall risk of bias (green = low risk; yellow = some concerns).

Study	Randomization	Deviations	Missing Data	Measurement	Selection	Overall
Pazhohan 2021 [14]	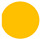	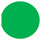	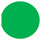	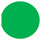	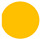	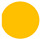
Nodler 2020 [15]	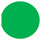	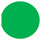	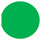	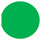	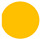	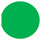
Santanam 2013 [18]	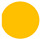	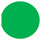	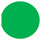	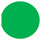	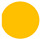	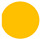
Mehdizadehkashi 2021 [13]	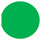	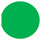	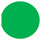	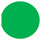	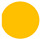	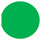
Almassinokiani 2016 [16]	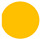	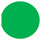	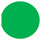	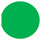	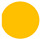	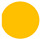
Amini 2021 [17]	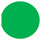	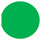	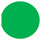	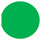	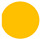	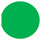
Mier-Cabrera 2008 [19]	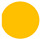	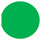	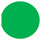	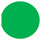	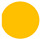	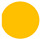

**Table 7:** Data extraction for vitamin D. Abbreviations: ↓, decrease; ↑ increase.

Study Reference	Year	Country	Type of Study	Sample Size	Participant Details	Age Range	Mean Age	Vitamin(s) Investigated	Formulation/Dosage	Intervention Duration	Control Condition	Outcome Measures	Key Results	Adverse Effects
Mehdizadehkashi et al. [13]	2021	Iran	Randomized, double-blind, placebo-controlled trial	60 (50 completed: 25 per group)	Women with confirmed endometriosis	18–40 years	≈35.2 ± 7 years	Vitamin D	50,000 IU vitamin D orally every 2 weeks	12 weeks	Placebo with identical appearance	Pelvic pain, lipid ratio, hs-CRP, TAC, glycemic and inflammatory markers	↓ Pelvic pain, ↓ hs-CRP, ↓ total/HDL cholesterol ratio, ↑ TAC; no effect on glycemic indices or other clinical symptoms	None reported
Pazhohan et al. [14]	2021	Iran	Randomized exploratory trial	34 (17 per group; 1 excluded from intervention due to pregnancy)	Women with stage III/IV endometriosis, infertile, regular cycles, BMI 18.5–30 kg/m^2^	22–37 years	≈30.2 ± 2.4 years	Vitamin D	50,000 IU orally per week (D-VIGEL 50000)	12–14 weeks	Routine hospital protocol (surgery + OCP)	Expression/activity of β-catenin (gene/protein), serum 25(OH)D, endometrial biopsy	↓ Active β-catenin protein, ↓ ratio of active/total β-catenin; no change in gene expression levels; ↑ serum 25(OH)D in intervention group	None reported
Nodler et al. [15]	2020	USA	Double-blind RCT, 3-arm parallel	69 (27 Vit D, 20 Fish Oil, 22 Placebo)	Adolescent girls and young women (12–25 y) with surgically confirmed endometriosis and pelvic pain	12–25 y	≈19.7 y	Vitamin D3 + Omega-3 FA	Vitamin D3: 2000 IU/day; Fish Oil: 1000 mg/day; Placebo: lactose capsule	6 months	Placebo (identical capsule)	Pain via visual analog scale (VAS); quality of life (SF-12); catastrophic thinking; pain medication usage; serum 25(OH)D and Omega-3 FA compliance	VAS pain ↓ in all groups; Vit D group had statistically significant ↓ pain (*p* = 0.02) but similar to placebo group (*p* = 0.07); Fish oil less effective. No statistical difference vs. placebo.	Minor (e.g., loose stools); most dropped due to personal reasons or follow-ups
Almassinokiani et al. [16]	2016	Iran	Double-blind randomized trial	38 women	Post-laparoscopy endometriosis patients	15–40 years	29.89 yrs	Vitamin D3	50,000 IU cholecalciferol weekly	12 weeks + follow-up to 24 wks post-op	Placebo	VAS scores for pelvic pain and dysmenorrhea	No statistically significant difference in pain scores between vitamin D and placebo groups at 24 weeks post-surgery	None reported

**Table 8:** Data extraction for vitamin C and E. ↓ decrease; ↑ increase.

Study Reference	Year	Country	Type of Study	Sample Size	Participant Details	Age Range	Mean Age	Vitamin(s) Investigated	Formulation/Dosage	Intervention Duration	Control Condition	Outcome Measures	Key Results	Adverse Effects
Amini et al. [17]	2021	Iran	Randomized, triple-blind, placebo-controlled clinical trial	60 (30 per group)	Women aged 15–45 years with laparoscopically confirmed stage I–III endometriosis	15–45 years	35.7 ± 5.71 years	Vitamin C and vitamin E	Vitamin C: 1000 mg/day (2 × 500 mg); vitamin E: 800 IU/day (2 × 400 IU)	8 weeks	Placebo tablets (mannitol, magnesium stearate, PVP)	Malondialdehyde (MDA), ROS, TAC levels; VAS for pelvic pain, dysmenorrhea, dyspareunia	↓ MDA and ROS; no change in TAC; significant ↓ in pelvic pain, dysmenorrhea, and dyspareunia compared to placebo	None reported
Santanam et al. [18]	2013	USA	Randomized, placebo-controlled clinical trial	59 (46 antioxidant, 13 placebo)	Women aged 19–41 years with pelvic pain and history of endometriosis and/or infertility	19–41 years	Not specified	Vitamin C and vitamin E	Vitamin E: 1200 IU/day (3 × 400 IU); vitamin C: 1000 mg/day (2 × 500 mg)	8 weeks	Placebo pills	VAS for chronic pelvic pain, dysmenorrhea, dyspareunia; ELISA for RANTES, IL-6, MCP-1 in peritoneal fluid	↓ Chronic pelvic pain (43%), ↓ dysmenorrhea (37%), ↓ dyspareunia (24%), ↓ inflammatory markers (RANTES, IL-6, MCP-1)	None reported
Mier-Cabrera et al. [19]	2008	Mexico	Randomized, double-blind trial	34 (16 vit group, 18 placebo)	Infertile women with stage I–II endometriosis confirmed via laparoscopy	25–35 years	≈32.7 ± 2.4 years	Vitamin C and vitamin E	Daily antioxidant bar: vitamin C 343 mg + vitamin E 84 mg	6 months	Identical placebo bar	Plasma/peritoneal levels of MDA and LOOHs; pregnancy rate	↓ Plasma MDA from month 4; ↓ LOOHs by month 6 in vit group vs. placebo; no change in peritoneal fluid markers; pregnancy rate ↑ (19% vs. 12%) but not statistically significant	None reported

## Data Availability

No new data were created or analyzed in this study. Data sharing is not applicable to this article.

## Supplementary Materials

The following supporting information can be downloaded at https://www.mdpi.com/article/10.3390/ijms27031476/s1. Reference [46] is listed in the Supplementary Materials.
